# Exploring differences in substance use behaviours among gender minority and non-gender minority youth: a cross-sectional analysis of the COMPASS study

**DOI:** 10.24095/hpcdp.44.4.04

**Published:** 2024-04

**Authors:** Thepikaa Varatharajan, Karen A. Patte, Margaret de Groh, Ying Jiang, Scott T. Leatherdale

**Affiliations:** 1 School of Public Health Sciences, University of Waterloo, Waterloo, Ontario, Canada; 2 Public Health Agency of Canada, Ottawa, Ontario, Canada; 3 Faculty of Applied Health Sciences, Brock University, St. Catharines, Ontario, Canada

**Keywords:** binge drinking, cannabis use, cigarette use, e-cigarette use, gender minority youth

## Abstract

**Introduction:**

Research characterizing substance use disparities between gender minority youth (GMY) and non-GMY (i.e. girls and boys) is limited. The aim of this study was to examine the differences in substance use behaviours among gender identity (GI) groups and identify associated risk and protective factors.

**Methods:**

Cross-sectional data from Canadian secondary school students (n=42107) that participated in Year 8 (2019/20) or Year 9 (2020/21) of the COMPASS study were used. Hierarchal logistic regression models estimated current substance use (cigarettes, e-cigarettes, binge drinking, cannabis and nonmedical prescription opioids [NMPOs]). Predictor variables included sociodemographics, other substances, mental health outcomes, school connectedness, bullying and happy home life. Interaction terms were used to test mental health measures as moderators in the association between GI and substance use.

**Results:**

Compared to non-GMY, GMY reported a higher prevalence for all substance use outcomes. In the adjusted analyses, GMY had higher odds of cigarette, cannabis and NMPO use and lower odds for e-cigarette use relative to non-GMY. The likelihood of using any given substance was higher among individuals who were involved with other substances. School connectedness and happy home life had a protective effect for all substances except binge drinking. Bullying victimization was associated with greater odds of cigarette, e-cigarette use and NMPOs. Significant interactions between GI and all mental health measures were detected.

**Conclusion:**

Findings highlight the importance of collecting a GI measure in youth population surveys and prioritizing GMY in substance use–related prevention, treatment and harm reduction programs. Future studies should investigate the effects of GI status on substance use onset and progression among Canadian adolescents over time.

**Highlights**

- Gender minority youth (GMY) were more likely to use cigarettes, cannabis and nonmedical prescription opioids and less likely to use e-cigarettes than girls and boys.
- GMY experiencing symptoms of depression or anxiety were less likely to binge drink than GMY without symptoms.
- GMY experiencing symptoms of anxiety were more likely to use nonmedical prescription opioids than GMY without symptoms.
- These findings support the need to prioritize GMY in substance use prevention programs.
- Youth surveillance studies should adopt the two-step gender identity measure.

## Introduction

Adolescence is a unique time in which individuals between the ages of 10 and 19 develop their gender identity (GI) and sexual orientation.^1^ According to the Survey of Safety in Public and Private Spaces, in 2018, individuals aged 15 to 24 years accounted for 30% of the lesbian, gay, bisexual, transgender, queer and Two-spirited (LGBTQ2+) population in Canada, as opposed to 14% of the non-LGBTQ2+ population.^2^ The term “gender minority youth” (GMY) refers to individuals whose GI is not cisgender (i.e. individuals whose GI corresponds with their sex assigned at birth [SAB]). GIs that fall under this umbrella term include, but are not limited to, transgender (i.e. someone whose GI does not match their SAB), nonbinary (i.e. a person whose GI is not limited to being exclusively male or female) and Two Spirit (i.e. an Indigenous person whose GI has both male and female spirits) populations.^3^

To date, GMY have been understudied in substance use research, as studies typically focus on the differences between cisgender boys and girls.^3^,^4^ This is because questions about GI have not yet been standardized on large-scale population-based surveys, thereby limiting the accuracy and inclusiveness of the data collected and mischaracterizing health and behavioural outcomes for GMY.^3^,^5^ Furthermore, many studies focussing on GMY are generally small-scale, lack comparison groups or fail to recognize that sexual orientation, SAB and GI are conceptually different.^3^,^5^,^6^ However, this is slowly changing, with national surveys adopting the two-step measure (Step 1 asks SAB; Step 2 asks current GI), as well as researchers, funders and journal editors emphasizing the need to examine the impacts of both sex and gender on health outcomes.^3^,^7^

Investigating substance use is essential, as the literature suggests that GMY are at a greater risk for substance use, misuse and related problems compared to cisgender youth.^4^,^8^-^12^ In 2017, findings from a cross-sectional study revealed that nonbinary Canadian youth (Grades 9–12) were 2.26 times more likely to ever use cannabis than males.^13^ A cross-sectional analysis of a sample of California youth (Grades 7–12) found that transgender youth had higher rates of lifetime, current and in-school substance use compared to non-transgender peers.^8^ Similarly, a national survey in the US highlighted that the rates of lifetime alcohol and past-30-day cigarette and cannabis use were higher among transgender youth than cisgender peers.^10^ Emerging evidence also anticipates GMY may have been disproportionately affected by the COVID-19 pandemic, thereby further exacerbating their risk for using substances.^14^

Substance use disparities among GMY may be explained by the minority stress theory, which postulates that GMY use substances to cope with the unique social stressors they experience in schools, families and communities as a result of their marginalized or stigmatized identities.^4^,^15^,^16^ The chronic stressors that impact their health and well-being may be external (distal) objective stressors (e.g. discrimination), proximal subjective stressors (e.g. hiding one’s GI), or both.^15^ The risk for problematic substance use may be further heightened among GMY who, in the absence of social support (e.g. support from school personnel), experience elevated rates of emotional dysregulation, social and interpersonal problems and psychological distress.^15^-^17^

Currently, the majority of research investigating GMY’s substance use behaviours stems from the US.^9^,^10^,^12^,^15^ Given the similar experiences with minority stressors, we expect Canadian GMY’s substance use patterns to mirror those in the US.^4^ Understanding substance use behaviours among Canadian GMY is critical in preventing adverse health and social outcomes and informing interventions efforts to effectively support the unique needs of this population. Thus, given the limited large-scale research among Canadian youth (aged 12–18),^13^,^18^ the purpose of this study was to (1) examine the differences in substance use behaviours between Canadian GMY and non-GMY, and (2) identify associated risk and protective factors. 

## Methods

***Ethics approval***

All procedures employed by the COMPASS study were approved by the University of Waterloo Office of Research Ethics (ORE #30118) and appropriate school board committees. 

***Procedure***

The COMPASS study is a prospective cohort study that annually collects data from full school samples of Canadian secondary school students (Grades 9–12, Secondary I–V in Quebec).^19^ Schools that permit an active-information passive consent parental permission protocol,^20^ which limits self-selection and response bias in substance use research, were purposefully sampled.^21^ A full description of the COMPASS study methods is available online (https://uwaterloo.ca/compass-system/about). 

Cross-sectional data from two consecutive waves (Year 8 [Y8]: 2019/20; Year 9 [Y9]: 2020/21) were used to increase the sample size among GMY. An anonymous, self-generated code was used to identity unique participants. Students were entered into the study once; for students that participated in both years, only their Y9 responses were used. Details on the data linkage process are described elsewhere.^22^ Data in Y8 were collected between September 2019 and February 2020 via the paper-based COMPASS Student Questionnaire, which was completed during class time.^23^ Since March 2020, when schools first suspended in-person learning due to COVID-19 restrictions, students have completed an online COMPASS Student Questionnaire2^4^ using Qualtrics XM25 survey software. 

Consistent with youth surveillance systems at the time of data collection,^5^,^26^,^27^ the COMPASS student questionnaire in Y8 and Y9 measured students’ GI with the question, “Are you female or male?” Response options included “female,” “male,” “I describe my gender in a different way” and “I prefer not to say (PNTS).” While the measure used enabled youth to identify with a GI outside the traditional binary categories, we recognize that by not specifying “sex” or “gender,” this question does not differentiate between youths’ SAB and current GI. Thus, the question could be construed as measuring students’ GI or biological sex.^28^,^29^

However, given that this study primarily focusses on the socially constructed roles, behaviours and identities of youth, we categorized students who responded “female” and “male” as “girl” and “boy,” respectively, (i.e. “non-GMY”). Students who responded, “I describe my gender in a different way” were categorized as “GMY.” We acknowledge that our definition of “non-GMY” does not meet the preferred cisgender classification. However, seeing that we do not have data for students’ SAB, we cannot definitively categorize youth as “cisgender.” Instead, we can utilize the existing gender measure to differentiate youth that do not self-identify with the conventional binary options from those that do, and provide further insight into the substance use disparities between groups—a topic on which there is a dearth of evidence. 

***Participants***

A total of 80608 students participated across 144 schools in Ontario, Alberta, British Columbia and Quebec. Students in Secondary I and II in Quebec (equivalent to Grades 7 and 8; n=20711) and students with missing values for any variable (n=17790; variables with missing values included gender [0.38%], cigarette use [6.0%], e-cigarette use[6.1%], binge drinking [5.4%], cannabis use[6.7%] and nonmedical prescription opioid use [NMPOU; 7.2%]) were excluded. Due to their unknown GI status, students who responded “PNTS” (n=570) for GI were excluded from regression analyses. However, some descriptive results comparing this group with girls, boys and GMY are provided.

Table 1 presents a chi-square analysis of demographic characteristics comparing students with missing outcome data versus complete data. Significant differences between groups were identified for all variables. The primary reasons for missing respondents were school absenteeism, spare study periods and parent refusals (<1%). The final complete-case analytic sample includes 41537 students attending 139 schools (Alberta, 3072; Ontario, 14626; Quebec, 16403; British Columbia, 7436).

**Table 1:** Chi-square analysis of demographic characteristics comparing students participating in Year 8 (2019/20) or Year 9 (2020/21) of the COMPASS study with missing outcome data versus complete data (N = 59 897)

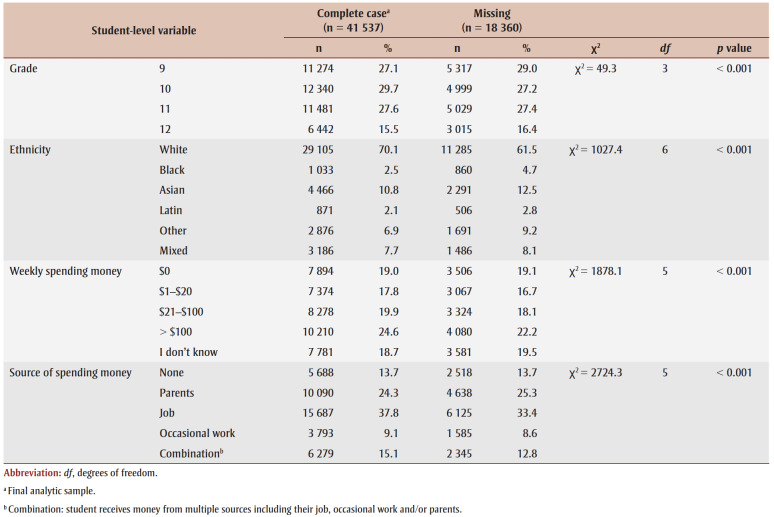

***Measures***

**Substance use**

Students reported on their cigarette use (“On how many of the last 30 days did you smoke one or more cigarettes?”); e-cigarette use (“On how many of the last 30 days did you use an e-cigarette?”); binge drinking (“In the last 12 months, how often did you have 5 drinks of alcohol or more on one occasion?”); and cannabis use (“In the last 12 months, how often did you use marijuana or cannabis? [a joint, pot, weed, hash]).” Students who reported past-month use were classified as current users and students who used less than once a month were classified as noncurrent users. NMPOU was assessed with the question, “Have you tried any of the following medications to get high?” with three medications listed: “oxycodone,” “fentanyl” and “other prescription pain relievers.” Responses were categorized into a binary variable; an answer of “Yes, I have done this in the last 12 months” to any of the three medications was classified as engaging in NMPOU in the past year.

**Mental health**

Self-reported past-week depression symptoms (e.g. negative affect, somatic symptoms and amotivation) were assessed using the 10-item Center for Epidemiologic Studies Depression Scale Revised (CESD-R-10).^30^ Students responded to items using a 4-point Likert scale (0=“none or <1 day” to 3=“5–7 days”). Sum scores were dichotomized, whereby a score of ≥10 signified students had clinically relevant symptoms of depression (henceforth referred to as “depression”).^30^ The CESD-R-10 items had an internal consistency of α=0.992.

The Generalized Anxiety Disorder 7-item (GAD-7) scale was used to measure self-reported symptoms of anxiety in the past two weeks.^31^ Students’ self-perceived feelings of worry, fear and irritability were rated using a 4-point Likert scale (0=“not at all” to 3=“nearly every day”). Sum scores were dichotomized, whereby a score≥10 denoted students had clinically relevant anxiety symptomology (henceforth referred to as “anxiety”).^31^ Internal consistency of GAD-7 items was high (α=0.991). 

Students’ self-rated psychosocial well-being (e.g. psychosocial prosperity, optimism and relationships) was measured using the Flourishing Scale.^32^ Students responded to 8 items using a 5-point Likert scale (0=“strongly disagree” to 4=“strongly agree”). Sum of the scores ranged from 8 to 40, where higher sum scores indicated greater well-being or flourishing. The Flourishing Scale had high internal consistency (α=0.995).

Emotional intelligence and regulation problems were assessed using a modified version of the Difficulties in Emotion Regulation Scale (DERS) in which one high-loading item from each of the six subscales was included, based on previous studies in adolescent samples.^33^-^36^ Total sum scores ranged from 6 to 30, with higher composite DERS scores indicating greater socioemotional dysfunction. Internal consistency of the DERS items was high (α=0.992). 

**Other covariates**

Students were asked, “In the last 30 days, in what ways have you been bullied by other students?” Responses were dichotomized, with “yes” indicating having been bullied (e.g. physical attacks, verbal attacks, cyber-attacks, damage to or theft of possessions) and “no” indicating not having been bullied.

School connectedness was measured using an adapted version of the National Longitudinal Study of Adolescent Health5-item scale,^37^ which asks students to indicate how strongly they agree or disagree with the following five statements: “I feel close to people atmyschool,” “I feel I am part of my school,” “I am happy to be at my school,” “I feel the teachers at my school treat me fairly” and “I feel safe in my school.” A sixth item, “Getting good grades is important to me” was added. A sum score ranging from 6 to 24 was developed, with higher sum scores indicating greater feelings of connectedness.

On a 5-point Likert scale, students rated how much they agreed or disagreed with the statement “I have a happy home life.” A response of 1 or 2 indicated students strongly agreed or agreed, respectively, that they had a happy home life. 

Students provided the following demographic information, which is consistent with other youth health research: grade; province; ethnicity (White, Black, Indigenous, Asian, Latin American, other, mixed); weekly spending money (none, $1–$20, $21–$40, $41–$100, >$100, don’t know); and source of money (I do not usually get any money, my parents/guardians, I get a paycheque from a job, I get paid cash for occasional work).

***Analysis***

All analyses were performed in SAS 9.4.^38^ Prevalence estimates and comparisons by GI were made using frequency tables and χ^2^ and one-way ANOVA tests. Intraclass correlation coefficients (ICCs) were calculated for each outcome variable, and modest to moderate amounts of within-school variation were detected (ICC_cigarette_=0.059; ICC_e-cigarette_=0.033; ICC_bingedrinking_=0.076; ICC_cannabis_=0.028; ICC_NMPO_=0.001), indicating that 0.1% to 7.6% of the variation in students’ substance use behaviours was due to school-level differences. Diagnostics assessing the risk of multicollinearity between potential explanatory variables revealed a minimal risk of collinearity, as none of the variance inflation factors exceeded 2. 

Binary logistic models that predict the log odds of cigarette use, e-cigarette use, binge drinking, cannabis use and NMPOU were built using generalized estimating equations via PROC GENMOD. Models for each outcome were built using a stepwise approach. Models I to IV added variables in the following order: gender, demographic characteristics, other substances and other covariates. Comparisons between GI groups were made by changing the reference group in the model. The moderating effects of all mental health variables were examined; each two-way interaction was tested in separate models. Comparisons between GI groups were assessed using the LSMEANS statement with the DIFF option.

## Results

***Student characteristics***

Table 2 presents the youths’ characteristics by GI. A small proportion of students identified as GMY (2.3%), while 51.8% identified as girls and 44.5% as boys. More youth participated in Y9 (n=29079) compared to Y8 (n=13028) of the COMPASS study; 75% of GMY participated in Y9. Although a majority of the participants identified as White (70%), half of GMY (49.9%) identified as an ethnicity other than White. A higher proportion of GMY reported having no weekly spending money relative to non-GMY. Students who preferred not to disclose their GI (1.4%) had similar characteristics to GMY. Significant differences for all covariates by GI were identified.

**Table 2:** Characteristics of high school students (N = 42 107; 139 schools) participating in Year 8 (2019/20) or Year 9 (2020/21) of the COMPASS study, by gender identity status

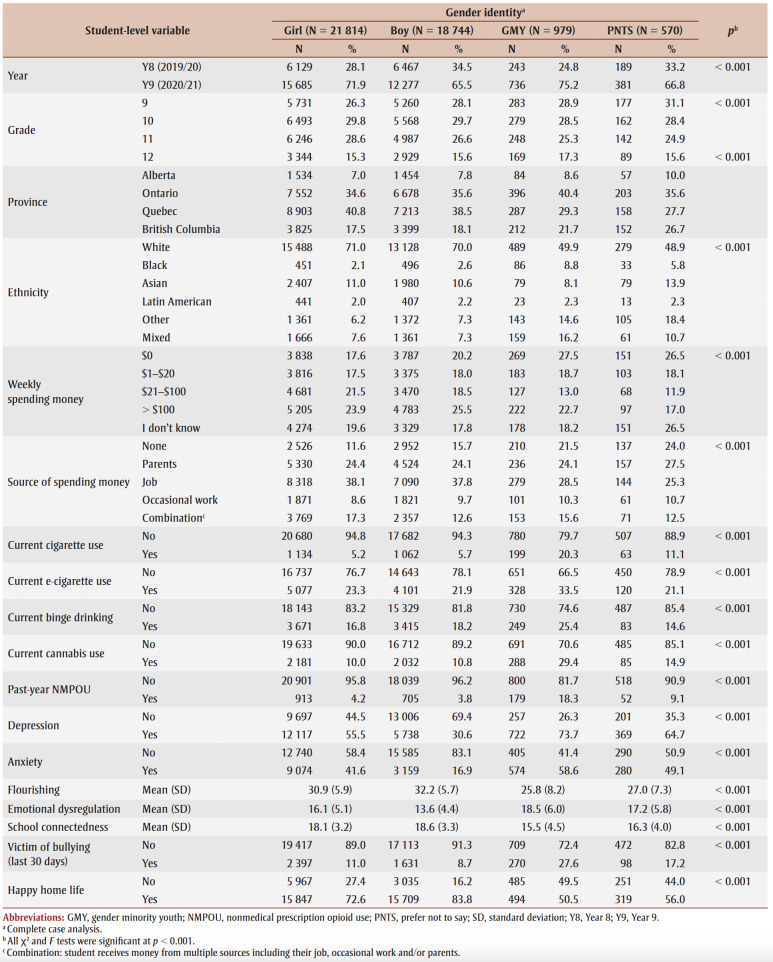

Compared to girls and boys, GMY had a higher prevalence of past-month use for all substances, with the use of cigarettes, cannabis and NMPOs being at least two to six times higher. Between girls and boys, the prevalence of substance use was similar. A substantially higher proportion of GMY, followed by girls, reported depression and anxiety compared to boys. On average, GMY reported lower mean flourishing and school connectedness scores and greater mean DERS scores than non-GMY. Boys had similar scores for flourishing and school connectedness as girls but had lower DERS scores. It should be noted that after GMY, students that did not disclose their gender status had the highest proportions of cigarette, cannabis and NMPO use and mental health and social problems.

***Predicting substance use***

Tables 3 and 4 present logistic regression results for cigarette use, e-cigarette use, binge drinking and cannabis use. Models I (unadjusted) and II (demographic-adjusted) indicate that GMY were more likely to engage in current substance use relative to non-GMY. After adjusting for concurrent substance use (Model III), cigarette, cannabis and NMPO use remained significant, with a positive association. 

**Table 3:** Generalized estimated equation models predicting the likelihood of substance use outcomes among high school students participating in Year 8 (2019/20) or Year 9 (2020/21) of the COMPASS study (N = 41 537)

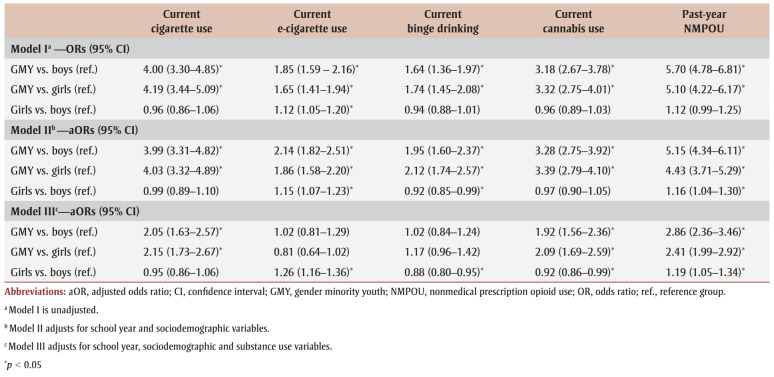

**Table 4:** Generalized estimating equation models predicting the likelihood of current substance use among high school students participating in Year 8 (2019/20) or Year 9 (2020/21) of the COMPASS study (N = 41 537)

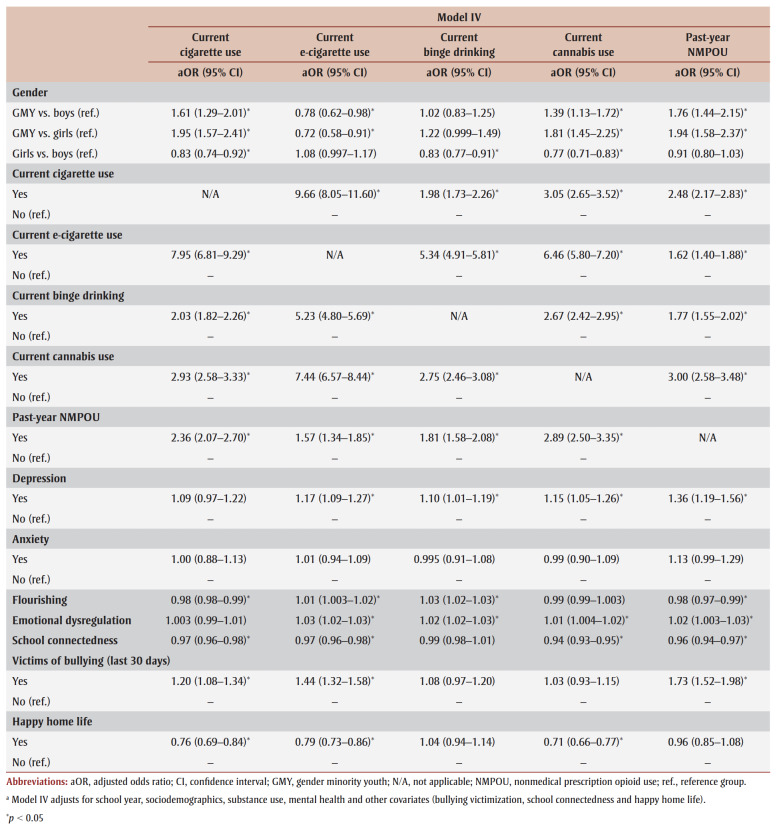

In the fully adjusted model (Model IV, which includes covariates), the adjusted odds ratio (aOR) was determined for each outcome. GMY had higher odds of using cigarettes (aOR_GMYvs.Boys_=1.61; aOR_GMYvs.Girls_= 1.95), cannabis (aOR_GMYvs.Boys_=1.39; aOR_GMYvs.Girls_=1.81) and NMPOs (aOR_GMYvs.Boys_= 1.76; aOR_GMYvs.Girls_=1.94) and lower odds of using e-cigarettes (aOR_GMYvs.Boys_=0.78; aOR_GMYvs.Girls_=0.72) than non-GMY peers. Girls had a lower likelihood of cigarette use (aOR=0.83), binge drinking (aOR=0.83) and cannabis use (aOR=0.77) compared to boys. Youth who used any of the substances were significantly more likely to use other substances. Prior to testing for interaction effects between mental health predictors and gender, youth with depression were 10% to 36% more likely to binge drink and use e-cigarettes, cannabis and NMPOs than those without depression. Anxiety had no significant effect on substance use. Although flourishing was associated with all substances (except cannabis) and DERS was related to every substance except cigarettes, the magnitude of the associations was small. 

School connectedness and happy home life were negatively associated with all substances except binge drinking. Students, on average, were 3% to 6% less likely to engage in substance use for every 1-point increase in school connectedness and 24% to 29% less likely if they reported having a happy home life. Youth who reported past-month bullying victimization had higher odds of using cigarettes (aOR=1.20), e-cigarettes (aOR=1.44) and NMPOs (aOR=1.73).

***Moderating effects of mental health predictors***

**Figure 1:**
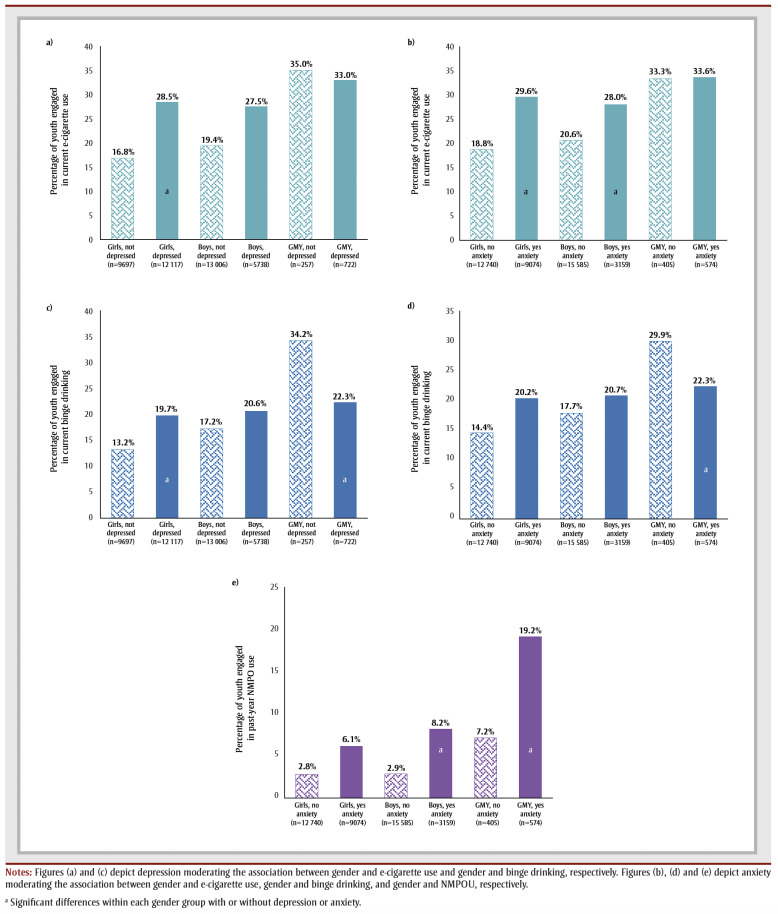
The percentage of youth reporting current e-cigarette use, binge drinking and nonmedical prescription opioid use (NMPOU) as a function of (1) gender depression and (2) gender anxiety

Overall, regardless of depression and anxiety status, a greater percentage of GMY compared to girls and boys reported e-cigarette use, binge drinking and NMPOU (Figure 1a–e). Depression was found to significantly moderate the association between gender and e-cigarette use and between gender and binge drinking. GMY with depression (22.3%) had a significantly lower prevalence of binge drinking compared to those without depression (34.2%, *p*<0.001; Figure 1c]. Comparatively, the prevalence of e-cigarette use and binge drinking was significantly higher for girls with depression than without (*p*<0.001; Figure 1a, c). 

Two-way interaction effects between gender and anxiety existed in e-cigarette use, binge drinking and NMPOU. GMY without anxiety had a significantly higher prevalence of binge drinking (29.9%) than GMY with anxiety (22.3%, *p*=0.005; Figure 1d). The proportion of girls and boys with anxiety using e-cigarettes was significantly higher compared to girls and boys without anxiety (*p*<0.05; Figure 1b). NMPOU was greater among GMY with anxiety (19.2%) than GMY without anxiety (7.2%; *p*=0.005; Figure 1e]. Boys with anxiety engaged in more NMPOU (8.2%) than boys without anxiety (2.9%; *p*=0.008; Figure 1e). Interaction effects between gender and flourishing and gender and DERS were significant for all outcomes except cigarette use. However, the estimates of the observed associations were small. Table 5 presents the two-way interaction effects.

**Table 5:** Generalized estimating equation models testing the moderating effects of mental health predictors on the relationship between gender identity status and substance use outcomes among a sample of high school students participating in Year 8 (2019/20) or Year 9 (2020/21) of the COMPASS study (N = 41 537)

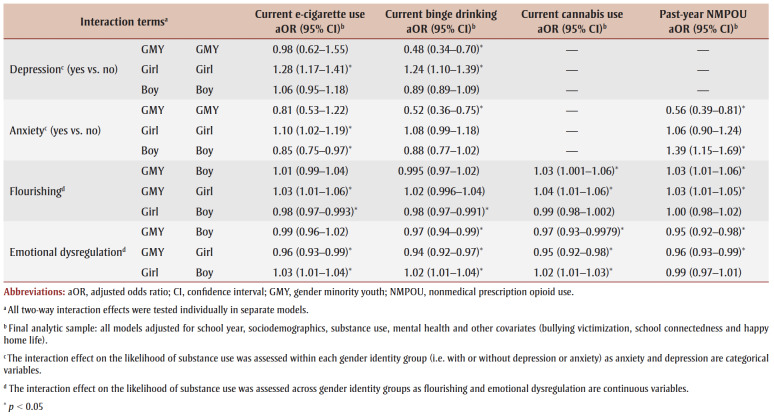

## Discussion

As expected from recent population studies surveying adolescents,^8^-^11^,^18^,^39^ the prevalence of substance use was higher among GMY than girls and boys. Interestingly, the frequency of substance use was also significantly higher among youth that indicated “PNTS” than girls or boys. It is possible that substance use among youth that reported PNTS may be driven by their own unique set of challenges (e.g. unsure about their GI).

Our results were consistent with De Pedro and colleagues’ cross-sectional study,^9^ which revealed higher rates of past-30-day cigarette and cannabis use among transgender youth compared to non-transgender peers. When adjusting for only sociodemographic characteristics, we found GMY had a higher likelihood of current e-cigarette use and binge drinking, similar to existing research.^9^,^39^,^40^ However, in our fully adjusted models, we found GMY relative to non-GMY had a lower likelihood of current e-cigarette use and that GMY status alone did not significantly predict current binge drinking. Our unique findings may be explained by the additional covariates (i.e. other substances, mental health outcomes, school connectedness, bullying victimization and happy home life) in our model and the relatively small difference in prevalence estimates between gender groups for e-cigarette use and binge drinking compared to the larger discrepancy seen for other substances.

Consistent with previous findings, we found that a greater proportion of GMY, followed by girls, reported mental health issues compared to boys.^8^,^41^,^42^ Interaction analyses indicated that the associations between gender and e-cigarette use, gender and binge drinking, and gender and NMPOU varied depending on mental health status. As expected, the frequency of NMPOU was greater among youth with clinically relevant anxiety symptoms than those without.^4^,^43^ Although GMY reported higher e-cigarette use and binge drinking compared to non-GMY, we found that binge drinking was lower among GMY with clinically relevant depression and anxiety symptoms than GMY without these conditions. This contradicts the current literature that suggests GMY experiencing internalizing symptoms will engage in greater substance use.^1^,^8^ E-cigarette use did not differ among GMY based on mental health status. However, for girls and boys, clinically relevant internalizing symptoms were associated with greater e-cigarette use, binge drinking and NMPOU. 

Additionally, and contrary to expectations,^16^,^44^ we did not find greater psychological well-being or poor emotional regulation skills to influence substance use among GMY. The insignificant findings may be because data were collected during the COVID-19 pandemic. The pandemic-induced lockdowns and restrictions, which upended youths’ daily routines, could have driven deteriorations in mental health and emotional dysregulation among all participating youth, regardless of their GI.^45^

A plausible explanation for our contradictory findings for binge drinking may be that GMY with internalizing symptoms are isolating themselves from social activities, in which binge drinking is common.^18^ For two-Spirit, lesbian, gay, bisexual, transgender, queer, intersex, and additional people who identify as part of sexual and gender diverse communities (2SLGBTQI+) youth, disclosing one’s sexual or gender identity has been linked to lower self-esteem, which is a prospective risk factor for depression and anxiety.^46^,^47^ If “coming out” is a positive experience, one in which youth feel accepted and supported by family, friends and community members, GMY may experience greater self-esteem and fewer internalizing symptoms, allowing them to better connect and socialize with peers.^18^,^46^,^47^ Future GMY-based research is needed to better understand the relationship between minority stress factors, mental health and substance use. 

This study, in line with existing research,^15^,^17^ also highlights that among the entire study sample, perceived happy home life and school connectedness had a protective effect against substance use, while bullying victimization was associated with an increased risk. Future work should examine the mechanisms underlying the association between social health factors and substance use among GMY.

***Strengths and limitations***

A primary strength of this study is that it is the first to use a large sample of Canadian secondary school students to examine differences in current substance use behaviours between GMY and non-GMY. The large sample size of youth is achieved via the robust COMPASS data collection procedures and data linkage process. Additionally, the GI measure was able to successfully capture GMY. 

Regarding the limitations of our study, first, our gender question does not identify the different subcategories of GMY (e.g. transgender, nonbinary). However, the proportion of GMY identified in our study (2%) aligns with other studies that sample youth attending secondary schools^48^ and is slightly higher compared to population-based studies that focus solely on transgender youth.^39^ Second, purposive sampling was used to recruit schools and collect data, which may limit the generalizability to school-aged youth in Canada. Third, the use of self-report measures (e.g. GI, substance use) may have led to underreporting due to social desirability bias. However, these risks were mitigated with the use of an anonymous, active-information, passive-consent data collection procedure that encourages participation as well as honest self-reporting.^20^,^21^ Fourth, the cross-sectional nature prohibits causal inferences. 

## Conclusion

We found significant disparities in substance use by GI, with GMY at a significantly greater risk of using some substances (i.e. cigarettes, e-cigarettes and NMPOs) compared to girls and boys. This study highlights the importance of adopting the two-step GI measure in population-based surveillance studies. Future studies should identify the longitudinal patterns of substance use behaviours by gender and sexual orientation status among Canadian adolescents. Such knowledge will be useful when implementing tailored community and school-based interventions that address the unique needs and challenges of GMY. 

## Acknowledgements

The COMPASS study has been supported by a bridge grant from the Canadian Institutes of Health Research (CIHR) Institute of Nutrition, Metabolism and Diabetes through the “Obesity – Interventions to Prevent or Treat” priority funding awards (OOP-110788; awarded to STL), an operating grant from the CIHR Institute of Population and Public Health (MOP-114875; awarded to STL), a CIHR project grant (PJT-148562; awarded to STL), a CIHR bridge grant (PJT-149092; awarded to KAP and STL), a CIHR project grant (PJT-159693; awarded to KAP) and by a research funding arrangement with Health Canada (#1617-HQ-000012; contract awarded to SL), a CIHR-Canadian Centre on Substance Use and Addiction team grant (OF7 B1-PCPEGT 410-10-9633; awarded to STL), aproject grant from the CIHR Institute of Population and Public Health (PJT-180262; awarded to STL and KAP).

A SickKids Foundation New Investigator Grant, in partnership with CIHR Institute of Human Development, Child and Youth Health (Grant No. NI21-1193; awarded to KAP) funds a mixed methods study examining the impact of the COVID-19 pandemic on youth mental health, leveraging COMPASS study data, and a CIHR Operating grant (UIP 178846, awarded to KAP) funds analysis of the impact of COVID-19 on health behaviours in COMPASS data.

The COMPASS-Quebec project additionally benefits from funding from the Ministre de la Sant et des Services sociaux of the province of Quebec, and the Direction rgionale de sant publique du CIUSSS de la Capitale-Nationale. TV is funded by the Ontario Graduate Scholarship (OGS) and by the Public Health Agency of Canada through the Federal Student Work Experience Program.

## Conflicts of interest

The authors declare that they have no conflicts of interest.

## Authors’ contributions and statement

TV—conceptualization, methodology, formal analysis, data curation, writing—original draft, review & editing. 

KAP—supervision, data curation, funding acquisition, resources, writing—review & editing. 

MdG—supervision, conceptualization, methodology, resources, writing—review & editing. 

YJ—supervision, conceptualization, methodology, resources, writing—review & editing. 

STL—supervision, data curation, funding acquisition, resources, conceptualization, methodology, investigation, writing—review & editing. 

All authors approved the final manuscript.

The content and views expressed in this article are those of the authors and do not necessarily reflect those of the Government of Canada.
